# Predicting the diagnosis of autism in adults using the Autism-Spectrum Quotient (AQ) questionnaire

**DOI:** 10.1017/S0033291716001082

**Published:** 2016-06-29

**Authors:** K. L. Ashwood, N. Gillan, J. Horder, H. Hayward, E. Woodhouse, F. S. McEwen, J. Findon, H. Eklund, D. Spain, C. E. Wilson, T. Cadman, S. Young, V. Stoencheva, C. M. Murphy, D. Robertson, T. Charman, P. Bolton, K. Glaser, P. Asherson, E. Simonoff, D. G. Murphy

**Affiliations:** 1 Department of Forensic and Neurodevelopmental Sciences, Institute of Psychiatry, Psychology & Neuroscience, King's College London, London, UK; 2 South London and Maudsley National Health Service Foundation Trust (SLAM), Maudsley Hospital, London, UK; 3 Child & Adolescent Psychiatry, Institute of Psychiatry, Psychology & Neuroscience, King's College London, London, UK; 4 Social, Genetic & Developmental Psychiatry (SGDP) Centre, Institute of Psychiatry, Psychology & Neuroscience, King's College London, London, UK; 5 Biological and Experimental Psychology, School of Biological and Chemical Sciences, Queen Mary University of London, London, UK; 6 Individual Differences, Language and Cognition Laboratory, Department of Developmental and Educational Psychology, University of Seville, Seville, Spain; 7 Centre for Mental Health, Imperial College London, London, UK; 8 Department of Psychology, Institute of Psychiatry, Psychology & Neuroscience, King's College London, London, UK; 9 Department of Social Science, Health & Medicine, School of Social Science & Public Policy, King's College London, London, UK

**Keywords:** Autism, autism spectrum disorders, neurodevelopmental disorders, screening, self-report

## Abstract

**Background:**

Many adults with autism spectrum disorder (ASD) remain undiagnosed. Specialist assessment clinics enable the detection of these cases, but such services are often overstretched. It has been proposed that unnecessary referrals to these services could be reduced by prioritizing individuals who score highly on the Autism-Spectrum Quotient (AQ), a self-report questionnaire measure of autistic traits. However, the ability of the AQ to predict who will go on to receive a diagnosis of ASD in adults is unclear.

**Method:**

We studied 476 adults, seen consecutively at a national ASD diagnostic referral service for suspected ASD. We tested AQ scores as predictors of ASD diagnosis made by expert clinicians according to International Classification of Diseases (ICD)-10 criteria, informed by the Autism Diagnostic Observation Schedule-Generic (ADOS-G) and Autism Diagnostic Interview-Revised (ADI-R) assessments.

**Results:**

Of the participants, 73% received a clinical diagnosis of ASD. Self-report AQ scores did not significantly predict receipt of a diagnosis. While AQ scores provided high sensitivity of 0.77 [95% confidence interval (CI) 0.72–0.82] and positive predictive value of 0.76 (95% CI 0.70–0.80), the specificity of 0.29 (95% CI 0.20–0.38) and negative predictive value of 0.36 (95% CI 0.22–0.40) were low. Thus, 64% of those who scored below the AQ cut-off were ‘false negatives’ who did in fact have ASD. Co-morbidity data revealed that generalized anxiety disorder may ‘mimic’ ASD and inflate AQ scores, leading to false positives.

**Conclusions:**

The AQ's utility for screening referrals was limited in this sample. Recommendations supporting the AQ's role in the assessment of adult ASD, e.g. UK NICE guidelines, may need to be reconsidered.

## Introduction

Autism spectrum disorders (ASD) are a family of lifelong neurodevelopmental syndromes with a prevalence of at least 1% (Baird *et al.* 2006; Baron-Cohen *et al.* 2009; Brugha *et al.* 2011*a*; Developmental Disabilities Monitoring Network Surveillance Year 2010 Principal Investigators, 2014). In the UK, it is estimated that each case of ASD is associated with average total lifetime costs of nearly £1 million (Buescher *et al.* 2014). Early and accurate diagnosis of ASD is crucial to enable affected individuals to receive treatment and support (Koegel *et al.* 2014). Nonetheless, ASD frequently goes undiagnosed into adulthood, which causes avoidable distress and morbidity (Geurts & Jansen, 2012; Nylander *et al.* 2013). Indeed, undiagnosed ASD is increasingly recognized as a significant problem in general adult psychiatry, and as an important differential diagnosis in this setting (Tebartz van Elst *et al.* 2013). Diagnosis of previously undetected ASD in adults is therefore key to managing the disorder, yet as there are no reliable biomarkers for ASD (Horder & Murphy, 2012), diagnosis relies on lengthy and costly expert clinical assessments. The capacity of adult ASD diagnostic services is, therefore, stretched.

In light of the fact that resource limitations can delay or prevent individuals from receiving a needed ASD assessment, it has been proposed that a means to ‘gate’ or triage referrals to such services is desirable. In particular, the Autism-Spectrum Quotient (AQ), a self-report questionnaire (Baron-Cohen *et al.* 2001), has been suggested as a means of quickly and cost-effectively estimating ASD risk and thus guiding referrals. The UK National Institute for Health and Care Excellence (NICE) recently endorsed the AQ for this purpose (Woodbury-Smith *et al.* 2005; NICE, 2012). NICE recommends that individuals with ‘possible autism’ should be offered a referral to a specialist diagnostic service if they score above the threshold (6 or more out of 10) on the AQ questionnaire.

However, it is not clear how well the AQ is suitable for the role of referral screening. While the AQ has been shown to discriminate well between individuals with diagnosed ASD and healthy controls (Baron-Cohen *et al.* 2001; Wakabayashi *et al.* 2006; Booth *et al.* 2013), little is known about whether the AQ can predict ASD diagnosis in adults with suspected ASD. One previous study (Ketelaars *et al.* 2008) found that the AQ was unable to predict ASD diagnosis within a clinically suspected population, although the sample size was small (21 ASD cases). Two other groups have reported that the AQ's performance in predicting ASD diagnosis was excellent (Woodbury-Smith *et al.* 2005) or fair (Sizoo *et al.* 2015). Yet no study to date has examined whether other psychiatric disorders (co-morbidities), such as anxiety or depression, act as confounds in the relationship between AQ scores and ASD diagnosis. This is a limitation, since co-morbidities are common in patients presenting for a diagnostic evaluation. Nor is it known whether the AQ predicts scores on the so-called ‘gold-standard’ (Baird *et al.* 2006) formal diagnostic assessments for ASD, the Autism Diagnostic Observation Schedule-Generic (ADOS-G) or the Autism Diagnostic Interview-Revised (ADI-R).

Therefore, we conducted the present study in order to, for the first time, examine the performance of the AQ questionnaire as a predictor of the outcome of an expert clinical ASD assessment, and the ADOS and ADI, in adults referred to a specialist diagnostic service with possible ASD, who were also assessed for co-morbidities. We studied a large sample of patients seen at a specialist diagnostic referral service. We examined the 10-item AQ10 version of the questionnaire (Booth *et al.* 2013), which is recommended in the NICE guidelines, and also the original 50-item version of the scale (AQ50). Our objective was to test the suitability of the AQ for ASD screening in the context prescribed by NICE.

## Method

### Recruitment

This study included 476 patients seen at the Behavioural Genetics Clinic (BGC) at the Maudsley Hospital in London, England. The BGC is a National Specialist Centre that accepts referrals from across the UK. The BGC offers a comprehensive psychiatric assessment; however, the focus is on the diagnosis of ASD, and the BGC only accepts referrals where the primary suspected diagnosis is ASD. The present sample represents all of the patients seen (consecutive admissions) at the BGC clinic over the period from September 2009 to May 2013. This is because, in September 2009 the BGC clinic added the AQ to the clinical questionnaire pack sent to all patients before their assessment, and in May 2013 the decision was taken to begin the current analysis of the AQ data. The only selection criteria were age 18+ years and giving informed consent. Over the period of this study, primary care (general practitioners) accounted for 37.7% of the referrals to the BGC, tertiary care 52.9%, and other sources 9.3%.

### Screening measures (AQ questionnaire)

Screening measures included the brief AQ10 questionnaire, and the full-length AQ50. Participants completed the AQ50, and we derived AQ10 scores from these answers, according to Allison *et al.* (2012). AQ10 scores are calculated by summing the following 10 items from the AQ50 questionnaire: items 5, 20, 27, 28, 31, 32, 36, 37, 41 and 45. These items were selected by Allison *et al.* (2012) on the basis that they best discriminated (i.e. have the highest discrimination index) between ASD patients and healthy controls. The original Autism-Spectrum Quotient (AQ50) contains 50 questions, relating to social skill, attention switching, attention to detail, communication and imagination. Each item has four possible responses (‘definitely agree’, ‘slightly agree’, ‘slightly disagree’, ‘definitely disagree’). Respondents are instructed to select one response per item. Items are scored dichotomously (i.e. collapsing ‘definitely agree’ and ‘slightly agree’ into ‘agree’ and similarly for ‘disagree’), with one point being assigned for each response characteristic of ASD. Total AQ50 scores therefore range from 0 to 50, with higher scores indicating more autistic traits. The published cut-off value for the AQ10 is ⩾6, i.e. scores of 6 or above are considered positive for ASD (Allison *et al.* 2012). For the AQ50, we compared two previously described cut-offs: the ‘clinical’ threshold of ⩾32 and the ‘screening’ cut-off of ⩾26 (Baron-Cohen *et al.* 2001). All questionnaires were administered in English.

Additionally, we investigated a novel variant of the AQ modified as an informant-rated scale. This measure, which has not been previously validated, was introduced in the BGC clinic to explore its potential as a non-self report measure of ASD symptoms and difficulties. A family member or close friend of the participant completed the informant AQ50. All of the questions were taken from the original AQ but re-worded to the gender-neutral third person. For example, AQ50 item 1: ‘I prefer to do things with others rather than on my own’ was changed to ‘S/he prefers to do things with others rather than on his/her own’. The informant AQ10 was derived in the same way as the self-report version. All questionnaires were administered in English.

### ASD assessment

Two formal ASD assessments are used in the BGC service: the ADOS-G (Lord *et al.* 2000) and the ADI-R (Lord *et al.* 1994). Experienced clinicians or clinical research workers administered these assessments. A clinical expert judgement (diagnosis) was made.

The ADOS-G is a measure of current ASD symptoms. The assessment comprises a structured activity session and semi-structured interview that includes a series of social presses and other opportunities intended to elicit behaviours associated with ASD. ADOS-G module 4, designed for verbally fluent adolescents and adults, was used.

The ADI-R is a parent/primary caregiver interview focused on developmental history of ASD-specific behaviours. The ADI-R is a measure of early-life symptoms. Scores are aggregated into symptom clusters that correspond to Diagnostic and Statistical Manual of Mental Disorders, 4th edition (DSM-IV) criteria for a diagnosis of autism (Lord *et al.* 1994).

Clinical diagnosis was made in a diagnostic meeting by consensus amongst a multidisciplinary team, including consultant psychiatrists, psychologists, psychiatry senior house officers (residents), specialist nurses and clinical research workers, using International Classification of Diseases (ICD)-10 research criteria and informed by the patient's ADOS-G and ADI-R scores. For this study, any autism-spectrum diagnosis was considered ‘ASD’, including diagnoses of autism and Asperger's syndrome.

Reflecting clinical practice at the BGC service, the ADI-R was always performed first if possible (i.e. if the parent or caregiver was alive and willing to be interviewed, and the patient provided consent for us to speak with them). If the ADI-R could not be performed, or if the outcome of the ADI-R was unclear, the ADOS-G was completed. Therefore some patients received an ADI-R only, some received an ADOS-G only, and a minority received both an ADI-R and an ADOS-G (these patients represented the more complex cases.)

### Sample size

A total of 476 adult patients were seen at the BGC clinic during the period of the study. Self-rated AQ50 scores were available for 456 of these (96%) and AQ10 scores were subsequently calculated for 428 (90%) (see online Supplementary Method). Of the patients, 473 (99%) received a clinical consensus expert diagnosis, 210 (44%) received an ADOS-G assessment, 305 (64%) received an ADI-R, of which 40 received both ADOS-G and ADI-R. No *a priori* sample size calculation was performed because these data were originally collected for clinical purposes.

### Supplementary information

For a further description of the assessment of other psychiatric disorders (co-morbidities), the BGC assessment procedures, data analysis and the treatment of missing items, and inclusion and exclusion criteria, please see the online Supplementary Method.

### Ethical standards

All procedures contributing to this work comply with the ethical standards of the relevant UK and institutional guidelines and with the Helsinki Declaration of 1975, as revised in 2008. All participants provided written consent to use outcome measures and clinical data for research purposes, and the study was approved by the National Research Ethics Service (NRES) Ethics Committee London – South East (ref: 12/LO/0790).

## Results

### Study population

The sample of 476 adults included 355 males (75%) and 121 females (25%). Age ranged from 18 to 70 years, with a median age of 29 years. Of the participants, 115 (24%) were aged 21 years or younger. Of the patients, 346 (73%) were assigned a diagnosis of ASD by the clinical team at the BGC (see Table 1).

**Table 1.** Patient characteristics

Measure	
Gender, *n* (%)	
Male	355 (75)
Female	121 (25)
Mean age, years (s.d.)	32.3 (11.4)
Mean AQ10 (s.d.)	7.2 (2.3)
Mean AQ50 (s.d.)	34.9 (8.2)
Clinical ASD diagnosis, *n* (%)	
Positive	346 (73)
Negative	126 (27)

s.d., Standard deviation; AQ, Autism-Spectrum Quotient; ASD, autism spectrum disorder.

### The AQ10 as a predictor of ASD diagnosis

The AQ10, with a cut-off of ⩾6, did not predict ASD diagnosis better than chance [χ^2^ = 1.423, degrees of freedom (df) = 1, *p* = 0.233]. While the test showed high sensitivity (0.77) as a predictor of receiving an ASD diagnosis, its specificity was less good (0.29) (see Table 2). The positive predictive value was high (0.76), indicating that three-quarters of those scoring ⩾6 on the AQ10 did receive a clinical diagnosis of ASD. However, the low negative predictive value (0.36) implies that nearly two-thirds of those who scored below the ⩾6 AQ10 cut-off, predicted not to receive an ASD diagnosis, in fact were diagnosed with ASD. For a *post-hoc* power analysis of these comparisons, see the online Supplementary Results.

**Table 2.** Diagnostic accuracy of the self-report and the informant-report AQ10 and AQ50

Predictor	Cut-off	*n*	Sensitivity (95% CI)	Specificity (95% CI)	PPV (95% CI)	NPV (95% CI)	Accuracy, %	Youden J^a^
Self-report AQ10	⩾6	425	0.77 (0.72–0.82)	0.28 (0.20–0.38)	0.76 (0.70–0.80)	0.30 (0.22–0.40)	65	0.05
Self-report AQ50	⩾26	453	0.88 (0.84–0.91)	0.20 (0.13–0.28)	0.76 (0.71–0.80)	0.36 (0.25–0.49)	70	0.07
Self-report AQ50	⩾32	453	0.71 (0.65–0.75)	0.35 (0.27–0.44)	0.76 (0.71–0.80)	0.29 (0.22–0.38)	61	0.06
Informant-report AQ10	⩾6	355	0.79 (0.73–0.84)	0.31 (0.22–0.41)	0.76 (0.72–0.81)	0.35 (0.25–0.46)	66	0.10
Informant-report AQ50	⩾26	381	0.93 (0.89–0.95)	0.14 (0.08–0.22)	0.74 (0.69–0.79)	0.41 (0.25–0.59)	71	0.07
Informant-report AQ50	⩾32	381	0.77 (0.72–0.82)	0.38 (0.28–0.48)	0.77 (0.72–0.82)	0.38 (0.29–0.48)	67	0.15

AQ10, 10-Item Autism-Spectrum Quotient; AQ50, 50-item AQ; CI, confidence interval; PPV, positive predictive value; NPV, negative predictive value.

a Youden J is a summary measure of the informative power of a test, and is defined as (sensitivity + specificity – 1) (Youden, 1950). A Youden J value of 0 means the test is uninformative and J of 1 indicates that the test is perfectly informative. 95% CIs are shown in parentheses for sensitivity, specificity, PPV and NPV.

We applied a receiver operating characteristic (ROC) curve analysis to evaluate the performance of the AQ10 with a range of cut-off criteria. The AQ10 did not significantly predict clinical diagnosis by this criterion either [area under the curve (AUC) 0.55, *p* = 0.12] (see online Supplementary Fig. S1).

**Fig. 1.**
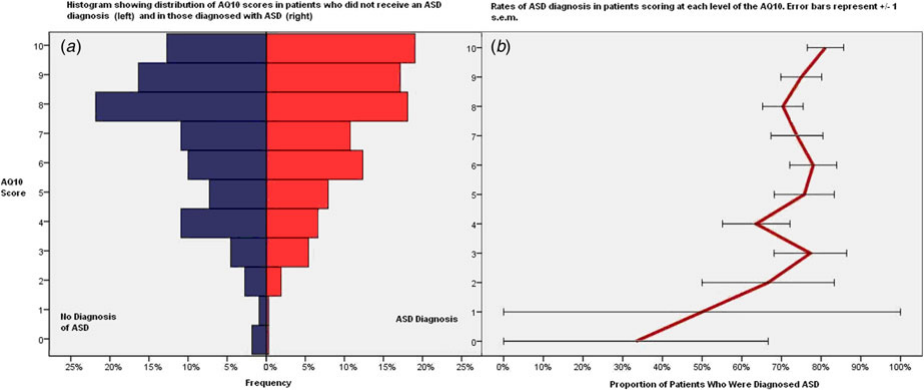
Distribution of 10-item Autism-Spectrum Quotient (AQ10) scores in patients according to whether they subsequently received a clinical diagnosis of autism spectrum disorder (ASD). (*a*) Histograms showing the proportion of patients who did not receive an ASD diagnosis (left) and of those who did receive a diagnosis (right) scoring at each level on the AQ10 (score out of 10). (*b*) Proportion of those scoring at each level of the AQ10 who received an ASD diagnosis. Values are means, with standard errors (s.e.m.) represented by horizontal bars. For a colour figure, see the online version.

Fig. 1 shows the distribution of AQ10 scores according to ASD diagnosis status. There was no significant difference in mean AQ10 scores between those diagnosed with ASD and those without ASD (ASD mean 7.26, s.d. = 2.28; not-ASD mean 6.85, s.d. = 2.46, *t* test, *p* = 0.12), confirming that AQ10 scores are not predictive of clinical diagnosis.

### Comparison of the AQ10 and AQ50 questionnaires

In a secondary analysis, we examined whether the original AQ50 questionnaire predicts ASD caseness better than the brief AQ10 (see Table 2). Applying the ‘screening’ cut-off of ⩾26, the AQ50 performed significantly better than chance (χ^2^ = 3.976, df = 1, *p* = 0.046). However, its predictive power was modest, including high sensitivity (0.88) but very low specificity (0.20). At the higher ‘clinical’ cut-off of AQ50 ⩾32, performance was no better than chance (χ^2^ = 1.265, df = 1, *p* = 0.261).

### Comparison of the AQ informant-report and self-report

The performance of the informant-report AQ50 and AQ10 was similar to that of the self-report versions of the scale (see Table 2). The AQ10 (χ^2^ = 3.658, df = 1, *p* = 0.056) and AQ50 cut-off ⩾26 (χ^2^ = 3.785, df = 1, *p* = 0.052) both predicted caseness marginally better than chance. The AQ50 cut-off ⩾32 was significantly superior to chance (χ^2^ = 8.860, df = 1, *p* = 0.003), but even here the specificity was poor (0.38). In the ROC curve analysis of the informant-report AQ50 and AQ10, these measures performed very similarly to the self-report versions. The informant AQ50, though not the AQ10, predicted clinical diagnosis slightly better than chance (AUC 0.58, *p* = 0.017), similarly to the self-report AQ50 (where the AUC was 0.56). The best performance was with a cut-off of ⩾32 at which there was a sensitivity of 0.78 and a specificity of 0.38, and an accuracy of 67% (see online Supplementary Fig. S2).

### Correlations between AQ and the ADOS-G and ADI-R

In order to address the question of whether AQ scores are predictive of the severity of ASD symptoms as continuous variables, we examined Pearson product-moment correlations between AQ scores and total scores on the ADOS-G and the ADI-R. ADOS-G totals were the sum of the communication, social, and repetitive/restricted behaviours domains. ADI-R totals were the sum of the communication, social, and repetitive domains. ADOS-G totals were not correlated with either the self-report AQ10 or the AQ50 (all *p* > 0.2). In contrast, ADI-R totals were correlated with the self-report AQ10 (*r* = 0.12, *p* = 0.045) and the AQ50 (*r* = 0.13, *p* = 0.030), albeit weakly (see Table 3). These results show that self-reported ASD traits, measured using the AQ, are not correlated with clinician-rated current ASD behaviours (ADOS-G), but are weakly associated with retrospectively reported early-life ASD symptoms (ADI-R). The correlation between ADOS-G and ADI-R scores was of trend significance (*r* = 0.29, *p* = 0.072, *n* = 40).

**Table 3.** Correlations amongst AQ scores, and total ADOS-G (communication + social + repetitive/restricted behaviours) and total ADI-R scores^a^

	Self-report AQ50	Self-report AQ10	Informant AQ50	Informant AQ10	ADOS-G total	ADI-R total
Self-report AQ50						
*r*	1	0.82**	0.56**	0.33**	0.06	0.13*
*p*		<0.001	<0.001	<0.001	0.433	0.030
*n*		428	364	340	204	290
Self-report AQ10						
*r*		1	0.47**	0.43**	−0.05	0.12*
*p*			<0.001	<0.001	0.459	0.045
*n*			341	321	194	271
Informant AQ50						
*r*			1	0.74**	0.038	0.37**
*p*				<0.001	0.644	<0.001
*n*				358	153	260
Informant AQ10						
*r*				1	−0.05	0.29**
*p*					0.583	<0.001
*N*					141	245
ADOS-G total						
*r*					1	0.29
*p*						0.072
*n*						40
ADI-R total						
*r*						1
*p*						
*n*						

AQ, Autism-Spectrum Quotient; ADOS-G, Autism Diagnostic Observation Schedule-Generic; ADI-R, Autism Diagnostic Interview-Revised; AQ50, 50-item AQ; AQ10, 10-item AQ.

a All correlation coefficients are bivariate Pearson's *r. p* Values are two-tailed. The number of observations (*n*) for each comparison varies because not all participants underwent all assessments.

Correlation significant: * *p* < 0.05, ** *p* < 0.001.

### Associations between psychiatric disorders (co-morbidities) and AQ scores

We obtained data on co-morbidities for 396 patients (84% of the sample). Across the whole sample, major depressive disorder was present in 20% of cases, panic/agoraphobia 12%, generalized anxiety disorder (GAD) 20%, social anxiety/phobia 17%, attention-deficit/hyperactivity disorder 16%, and obsessive–compulsive disorder 20%. Overall, 69% of patients were diagnosed with one or more co-morbidities (see Table 4).

**Table 4.** Rates of co-morbidities in true and false positives, and true and false negatives, with caseness defined by ASD clinical diagnosis and screening prediction of ASD defined as AQ10 self-report scores of ⩾6

	Rate of disorder in AQ10 true positives (*n* = 201)	Rate of disorder in AQ10 false positives (*n* = 71)	Rate of disorder in AQ10 true negatives (*n* = 27)	Rate of disorder in AQ10 false negatives (*n* = 59)
Depression, %	21.9	21.1	14.8	18.6
Panic/agoraphobia, %	17.9	2.8	7.4	10.2
Generalized anxiety disorder, %	24.9	21.1	3.7	15.3
Social anxiety, %	18.4	16.9	14.8	15.3
Attention-deficit/hyperactivity disorder, %	15.9	14.1	22.2	11.9
Obsessive–compulsive disorder, %	26.9	9.9	14.8	13.6
Any co-morbidity, %	76.1	57.8	66.7	61.0

ASD, Autism spectrum disorder; AQ10, 10-item Autism-Spectrum Quotient.

Compared with true negatives (who scored <6 on the AQ10 and did not receive a clinical ASD diagnosis), false positives (<6 on the AQ10 but did receive a diagnosis) were more likely to have GAD (21.1% *v.* 3.7%, χ^2^ = 4.347, df = 1, *p* = 0.037). This suggests that GAD might ‘mimic’ ASD and lead to false positives on the AQ. In contrast, false negatives were less likely to have at least one co-morbidity than true positives (61.0% *v.* 76.1%, χ^2^ = 5.241, df = 1, *p* = 0.022). This suggests that individuals with ‘pure’ ASD, and no co-morbidities, might be more likely to be missed by the AQ questionnaire. See Table 4 for co-morbidity rates for each of the four groups (true positive, false positive, true negative, and false negative).

To discover whether co-morbidities predict AQ scores independent of ASD caseness, we performed a general linear model (GLM) analysis with AQ10 scores as the dependent variable. Independent variables (fixed-effect factors) were: clinical ASD diagnosis, and the six co-morbidities as binary predictors. The GLM revealed that GAD (*p* = 0.014) was the only significant predictor of AQ10 scores, while ASD diagnosis did not predict AQ10 scores (*p* = 0.537) in this analysis. See online Supplementary Table S1 for the full GLM results. The AQ questionnaire therefore seems to be more sensitive to the presence of GAD than it is to ASD in this sample. Those individuals with ASD who present with no psychiatric co-morbidities may be at risk of scoring below threshold on the AQ and thus becoming false negatives if the AQ is used as a screening tool.

### Financial implications of using the AQ to triage referrals

In order to put these results into a financial context, we estimated the financial implications had the BGC clinic adopted the NICE guidelines suggesting that referrals for ASD assessment should be made for a score of ⩾6 on the AQ10. This does not represent a comprehensive health economics analysis, as we do not attempt to estimate the value of receipt of an ASD diagnosis to an adult with ASD, or the cost to society of a case of undiagnosed ASD.

In our sample of consecutive BGC patients spanning 4 years, 102 (24%) scored 5 or below on the AQ10. Given that each BGC assessment costs the National Health Service (NHS) £2305 on average, according to a 2011 estimate (Murphy *et al.* 2011), this implies that a total of £ 235 110 (102 × £2305) could have been saved if all of these ‘AQ10 negative’ individuals had not received a BGC referral, assuming that all other factors remained equal. However, 71 of those 102 referrals went on to receive a clinical diagnosis of ASD at the BGC, that is, 70% of the ‘negatives’ proved to be false negatives. Therefore, for every 10 patients denied a referral, £ 23 050 would have been saved but seven ASD cases would go undiagnosed. For every £3300 (£ 235 000/71) saved, an individual with ASD would miss out on a diagnosis.

Regarding the issue of false positives, of the 425 referrals, 323 (76%) scored 6 or above on the AQ10 and would have been offered a referral under the NICE guidelines. However, of these 323, 79 (24%) were diagnosed as not having an ASD at the clinical assessment. As each assessment costs £2305, these false positives on the AQ10 would have cost the NHS £ 182 095 (79 x £2305) over the 4 years of the study, or about £ 45 000 per year. Some of these patients may have benefited from their BGC assessment despite not receiving an ASD diagnosis, e.g. by receiving a diagnosis of another psychiatric disorder. However, 30 of the 79 (38%) AQ10 ‘false positives’ were not assessed as having any psychiatric disorder.

## Discussion

We investigated the AQ questionnaire as a predictor of ASD caseness in a large sample of adults referred to our diagnostic clinic with suspected ASD. Our objective was to determine if the AQ would be an effective means of ‘gating’ clinical referrals, as recommended by the UK NICE guidelines (NICE, 2012). We found little evidence that the AQ could predict who would receive a clinical diagnosis of ASD in our sample. The brief AQ10 questionnaire was no better than chance as a predictor of ASD diagnosis, providing high sensitivity (0.77) but low specificity (0.29). Nearly two-thirds of the patients who scored below the cut-off score of 6 were ‘false negatives’, i.e. they went on to receive a diagnosis of ASD. The longer version of the questionnaire, the AQ50, performed only marginally better.

These results contrast with some prior findings. In two large studies (Allison *et al.* 2012; Booth *et al.* 2013), the AQ10 was found to discriminate well between ASD adults and healthy controls. However, Brugha *et al.* (2011*b*) found that it only modestly predicted ASD caseness within a large general population sample. In that study, the AQ had a sensitivity of 0.73 and a specificity of 0.62 when acting as a predictor of scoring above the threshold of 10 points on the ADOS-G, and AQ scores were significantly, but weakly, correlated with ADOS-G total symptoms (*r* = 0.24). In our sample we found a comparable sensitivity, but a substantially lower specificity, and no significant correlation between the ADOS-G and the AQ.

Why does the ability of the AQ to predict ASD diagnosis vary across studies? One plausible explanation is differences in the nature of the non-ASD group. The AQ shows excellent performance (Allison *et al.* 2012; Booth *et al.* 2013) in distinguishing ASD cases from healthy controls recruited from the general population. However, such case–control studies are known to overestimate the accuracy of diagnostic tests (Lijmer *et al.* 1999; Rutjes *et al.* 2006). Our study did not use a case–control design, and our results suggest that the AQ differentiates poorly between true cases of ASD, and individuals from the same clinical population who do not have ASD (Simonoff *et al.* 2008; Freeth *et al.* 2012; L Underwood *et al.*, unpublished observations). Furthermore, we show that scores on the AQ were not correlated with current ASD symptoms as measured with the ADOS-G. AQ scores did predict scores on the ADI-R, a retrospective measure of childhood symptoms, but only weakly.

If the AQ, in its current form, is poorly predictive of ASD diagnosis in a clinical setting, why is this? One possible explanation is that individuals with ASD lack insight into their own behaviour (Bishop & Seltzer, 2012) and so find it difficult to self-report their own symptoms. However, against this hypothesis, we found that even when an informant completed the AQ, scores only marginally predicted clinical diagnosis, and did not correlate with ADOS-G symptoms. While the informant AQ was more predictive of ADI-R scores, this may reflect the fact that the ADI-R is itself an informant measure and the same informant completed both.

Therefore, a lack of self-insight may not fully account for the frequent discrepancies between the AQ and other ASD measures. Co-morbidities may go some way to explain this issue. We found that the presence of ASD is not an independent predictor of AQ10 scores but GAD does predict higher AQ10 scores. Thus, GAD may contribute to false positives. We speculate that the AQ may be sensitive to anxiety because several AQ items take the form of self-evaluations, e.g. ‘I find it difficult to work out people's intentions’. Anxious individuals commonly lack confidence in their social abilities (Mathews & MacLeod, 2005) and it is possible that this low self-esteem might manifest as ASD-typical answers. Clinicians performing an ASD assessment, however, are able to use their judgement to distinguish between ASD and other conditions that can mimic ASD symptoms. We also showed that ASD individuals without co-morbidities tended to score low on the AQ, perhaps putting them at risk of becoming ‘false negative’ cases, those who had ASD but scored <6 on the AQ10.

Our results have implications for both clinical services and for future research. From a clinical perspective, we suggest that the adoption of the AQ as a clinical tool (as recommended by NICE) (NICE, 2012) will need to be guided by an assessment of the costs and benefits in relation to the particular patient population in which its use is proposed. We have shown that, in a population with suspected ASD seen at a national specialist diagnostic clinic, the sensitivity of the AQ is acceptable, but the specificity is poor, and the overall performance is little better than chance. As regards implications for future research, our findings highlight the need for measures of ASD symptoms that are suitable for use in the population with suspected ASD. It is possible that a subset of AQ50 items might be more effective than the AQ50 or the AQ10 subset that we examined. Alternatively, another ASD trait questionnaire might prove more suitable, for instance the Autism Spectrum Disorders in Adults Screening Questionnaire (ASDASQ) (Nylander & Gillberg, 2001). Finally, a novel scale more predictive of ASD diagnosis might be developed through a study of how clinicians discriminate ASD from ‘ASD-like’ symptoms.

Our study has a number of limitations. First, our study population was limited to one clinic, the BGC, which is a national referral centre. It remains to be determined how well our results would generalize to other clinical services. It is possible, for instance, that the AQ might perform better in screening the referrals received by smaller local ASD services, which receive a higher proportion of referrals from primary and secondary care. Future research should explore this possibility. Next, a limitation of this retrospective study was that we did not produce a published study protocol, and no *a priori* power calculations were performed. This was a retrospective analysis, rather than a prospective study designed to address a particular question. A further limitation is that we did not obtain intelligence quotient (IQ) scores for our participants. IQ has been shown to correlate with AQ scores (Bishop & Seltzer, 2012) but may be negatively associated with ADOS severity (Klin *et al.* 2007); therefore it would have been desirable to measure IQ as a potential confound. Similarly, while we had qualitative measures of psychiatric co-morbidity, i.e. ICD-10 diagnoses, we lacked continuous measures of the severity of these symptoms and so could not address these as confounds in a quantitative manner. We calculated the AQ10 scores by summing the appropriate items from the AQ50, rather than by administering the AQ10 as a questionnaire *per se*. It is possible that our results would be different had we done so. A final limitation is that we only examined the ADOS-G and ADI-R as ASD diagnostic tools, and did not evaluate other instruments, such as the Diagnostic Interview for Social and Communication Disorders schedule (Wing *et al.* 2002).

In conclusion, we report that the AQ did not predict ASD caseness in a large sample of patients seen at an adult ASD diagnostic service. Therefore, the utility of the AQ for triaging ASD referrals in those with suspected ASD is called into question. Being diagnosed with ASD can represent a turning point for patients – and 85% of UK adults diagnosed with an ASD were glad to receive the diagnosis (Jones *et al.* 2014). These results suggest that, in the diagnosis of ASD, self-report questionnaire measures may not yet be able to substitute for specialist clinical assessments.

## Supplementary material

For supplementary material accompanying this paper visit http://dx.doi.org/10.1017/S0033291716001082. click here to view supplementary material
