# Supplementary material for: Predicting the diagnosis of autism in adults using the Autism-Spectrum Quotient (AQ) questionnaire
Source: Psychol Med. 2016 Jun 29;46(12):2595–604. doi: 10.1017/S0033291716001082 (PMC4988267; doi:10.1017/S0033291716001082)
Supplement: Supplementary file 1 [file S0033291716001082sup001.doc]

**Contents of the Supplementary material**

A) Supplementary Method – pages 1–3

B) Supplementary Results – pages 4–5

C) Supplementary Figs S1 and S2 – pages 6–7

D) Supplementary Table S1 – page 8

E) Supplementary Table S2 – pages 9–10

**Supplementary Method**

Assessment Procedure and Missing Items

The AQ50 questionnaire (from which AQ50 and AQ10 scores were derived) was obtained for all participants, as part of the questionnaire pack sent to all patients in the BGC clinic. In the majority of cases the AQ50 was completed before the ADOS-G/ADI-R interviews took place. The AQ, ADOS-G, and ADI-R were scored independently, to ensure that scores on one measure would not influence the scoring of the other instruments.

We only calculated AQ10 scores when all ten of the required items were completed, considering it impossible to accurately correct for missing values for such a small pool of items. In the case of the AQ50, questionnaires with 10% or more blank or improperly completed items (5+ items) were discarded. AQ50 questionnaires with 1 - 4 missing items were corrected as follows: corrected score=raw score x (50 / (50 – missing items). This is equivalent to imputing missing responses as the mean of completed responses. 85 of the 456 AQ50s (19% of the total) were corrected in this way.

Exclusion Criteria

All individuals who completed the AQ50 as part of the questionnaire pack, and who were seen at the BGC, were included in the study, unless they: i) did not attend enough of the assessment process to provide both AQ scores and to receive a clinical expert diagnosis; or if they ii) declined consent to have their anonymised data used in research. Consent for research was obtained at the beginning of the assessment process. Individuals who did consent were given a further opportunity to opt out at a later date; or if they iii) Were aged below 18 years of age at the time of the assessment.

Measures of Psychiatric Disorders (Comorbidities)

Comorbid mental health problems are common in adults with ASD. To investigate and control for the presence of these disorders, we examined records from the comprehensive psychiatric interview (based on the DSM-IV) which forms part of the assessment process for all patients seen at the Behavioural Genetics Clinic. These assessments were conducted by an experienced psychiatrist.

For the purposes of this study we examined the following six disorders i) major depressive disorder (MDD), ii) panic disorder and/or agoraphobia, and iii) generalised anxiety disorder (GAD) iv) social anxiety disorder and/or social phobia v) attention-deficit hyperactivity disorder (ADHD) vi) obsessive compulsive disorder (OCD). Other disorders such as schizophrenia, substance abuse and personality disorders were diagnosed in only a small number of participants (10 or fewer), precluding statistical treatment, so we did not include them in this analysis.

We coded a disorder as 'present' if the patient was given a current definite diagnosis of the disorder at the assessment. Each of the six disorders was coded as a separate binary variable where 0=absent, 1=present. No hierarchy rules were applied.

Data Analysis and Statistics

The data was analysed using SPSS 21. To examine the performance of the screening measures (see above), we tested the performance of the AQ10 and AQ50 screening measures as a predictor of clinical diagnosis. To determine the statistical significance of the AQ10 and AQ50, we used chi squared tests with scoring above threshold vs. receiving a clinical diagnosis, both as binary variables. We further examined test performance using the following metrics: accuracy (% of predictions that were correct), sensitivity (correct positives / total positives), specificity (correct negatives / total negatives), positive predictive value (PPV), negative predictive value (NPV), and Youden’s J (Youden 1950), a measure of the information content of the test, equal to: Sensitivity + Specificity – 1. J ranges from -1 to 1, with 0 indicating that the test provides no useful information. The values of accuracy, sensitivity, specificity were interpreted as follows: values <0.70 are ‘poor’; 0.70-0.79 ‘fair’; 0.80-0.89 ‘good’; 0.90-1.00 ‘excellent’ (Cicchetti et al. 1995). These guidelines have been used previously when evaluating the properties of ASD screening tools (Oosterling et al. 2009).

To further investigate the psychometric properties of the AQ at a range of cut-offs, we calculated Receiver Operating Characteristic (ROC) curves for i) the AQ10 and ii) the AQ50 as predictors of clinical judgement. The outcome was the Area Under the Curve (AUC), which is 0.5 under the null hypothesis of no significant predictive power at any cut-off. If the AUC was significantly different from 0.5, the optimal cut-off and the Youden’s J at that cut-off were calculated. For the presence of other psychiatric diagnoses, as these data were nominal, chi-squared tests were used in order to determine whether rates of each comorbidity differed across different participant groups (e.g. those receiving a clinical diagnosis of ASD vs. those not receiving one.)

**Supplementary Results**

Comparison of the ADOS-G and ADI-R As Definitions of Caseness

To determine whether AQ scores predict scoring above the ASD threshold on the ADOS-G and ADI-R criteria, we repeated our primary analyses using ADOS-G and ADI-R scores to define caseness in place of clinical consensus diagnosis. We applied the standard, validated cut-offs (ADOS-G: communication ≥ 2 and social ≥4 and social + communication ≥ 7. ADI-R: social ≥ 10 and communication ≥ 8 and repetitive behaviours ≥ 3.) See Supplementary Table S2. With the ADOS-G, no variant of the AQ predicted caseness better than chance (all χ^2^ < 0.759, all p > 0.384). For ADI-R caseness, scoring ≥32 on the self-report AQ50 predicted caseness (χ^2^=7.071, df=1, p=0.014) however the self-report AQ10 did not. The informant-rated AQ50 was predictive of the ADI-R, with the AQ50 ≥26 (χ^2^=7.708, df=1, p=0.005) and ≥32 (χ^2^=15.662, df=1, p<0.001) although the AQ10 did not significantly predict it. In summary, the AQ is poorly predictive of clinical diagnosis and of ADOS-G caseness. There is some evidence that the AQ50, especially the informant-report version, may predict ADI-R caseness, but not the AQ10.

Post-hoc Power Analysis

As this was a retrospective study, no a priori power calculation was performed. However, we calculated the post hoc power for our key comparisons. The key comparisons take the form of chi-squared tests with *scoring above threshold on the AQ* as the row variable and *receiving a* *clinical diagnosis of ASD* as the column variable.

For the test of the predictive ability of scoring 6+ on the self-report AQ10, we found that 72% of the non-ASD group tested positive. We had 80% power to detect a positive test rate of >86% (or less than 57%) in the ASD group, i.e. we had 80% power to detect an absolute 14% increase in positive AQ10 scores in the ASD group compared to the non-ASD group. For the self-report AQ50, 80% of non-ASD scored 26+ and we had 80% power to detect a rate of positive scores of >92% (or less than 66%) in the ASD group, an absolute 12% increase. For the self-report AQ50 32+ the non-ASD positive rate was 65% and we had 80% power to detect >80% (or less than 49%) an absolute 15% increase.


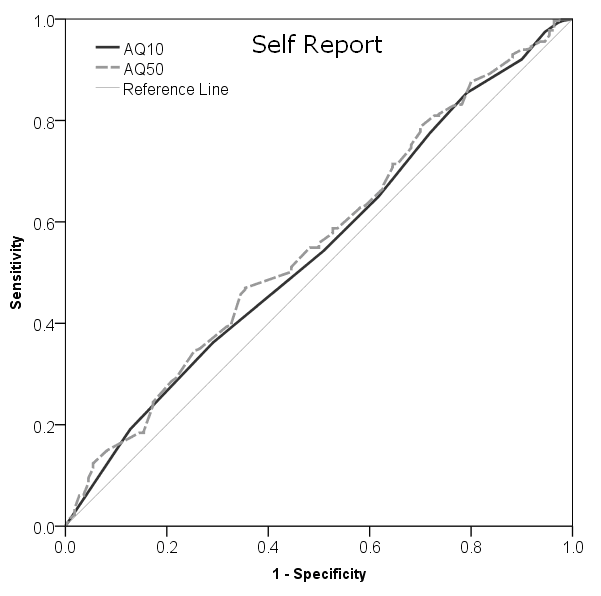


**Supplementary Fig. S1.** ROC Curve showing performance of the self-report AQ10 and AQ50 questionnaires as predictors of ASD clinical diagnosis. The grey diagonal line represents the line of chance performance (Sensitivity = 1 – Specificity).


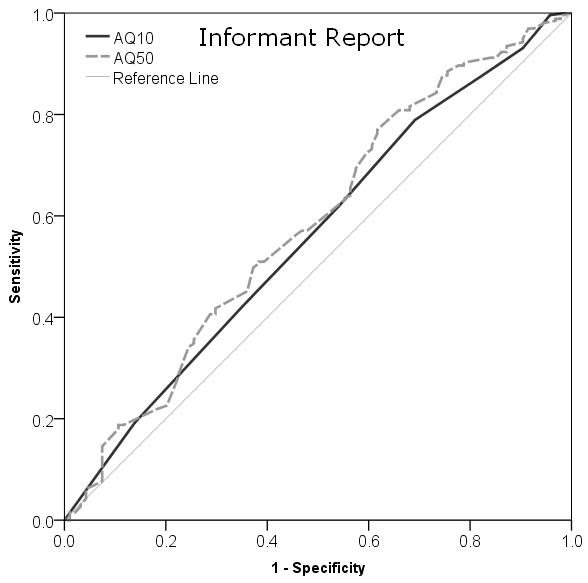


**Supplementary Fig. S2.** ROC Curve showing performance of the informant-report AQ10 and AQ50 questionnaires as predictors of ASD clinical diagnosis. The grey diagonal line represents the line of chance performance (Sensitivity = 1 – Specificity).

| Dependent Variable: AQ10 self report | | | | | |
| --- | --- | --- | --- | --- | --- |
| Source | Type III Sum of Squares | df | Mean Square | F | Sig. |
| **Corrected Model** | **94.772^a^** | **7** | **13.539** | **2.640** | **.011** |
| Intercept | 4193.372 | 1 | 4193.372 | 817.666 | .000 |
| Depression | 12.314 | 1 | 12.314 | 2.401 | .122 |
| Panic / Agoraphobia | 10.150 | 1 | 10.150 | 1.979 | .160 |
| **GAD** | **31.489** | **1** | **31.489** | **6.140** | **.014** |
| Social Anxiety | .509 | 1 | .509 | .099 | .753 |
| ADHD | 2.988 | 1 | 2.988 | .583 | .446 |
| OCD | 8.414 | 1 | 8.414 | 1.641 | .201 |
| Clinical ASD  Diagnosis | 1.963 | 1 | 1.963 | .383 | .537 |
| Error | 1794.962 | 350 | 5.128 |  |  |
| Total | 20153.000 | 358 |  |  |  |
| Corrected Total | 1889.735 | 357 |  |  |  |
| a. R Squared = .050 (Adjusted R Squared = .031) | | | | | |

**Supplementary Table S1.** SPSS 21 regression results of the general linear model (GLM) analysis with AQ10 scores as the dependent variable, and six comorbidities and clinical ASD diagnosis as binary predictor, in a fixed effects model. Significant predictors are highlighted in bold. The overall (corrected) model is significant, but only GAD (generalized anxiety disorder) is a significant predictor of AQ10 scores.

| Predictor | AQ  Cut-Off | Caseness  Criterion | N | Sensitivity  (95%  CI) | Specificity  (95%  CI) | PPV  (95% CI) | NPV  (95% CI) | Acc | Youden  J |
| --- | --- | --- | --- | --- | --- | --- | --- | --- | --- |
| Self-Report  AQ10 | >=6 | ADOS-G | 194 | 0.80  (0.72-0.86) | 0.25  (0.15-0.37) | 0.66  (0.58-0.73) | 0.40  (0.26-0.57) | 60% | 0.05 |
|  |  | ADI-R | 271 | 0.80  (0.70-0.87) | 0.29  (0.23-0.37) | 0.38  (0.31-0.45) | 0.73  (0.61-0.83 | 47% | 0.09 |
| Self-Report  AQ50 | >=26 | ADOS-G | 204 | 0.87  (0.80-0.92) | 0.13  (0.06-0.23) | 0.65  (0.58-0.72) | 0.35  (0.18-0.56) | 60% | 0.00 |
|  |  | ADI-R | 290 | 0.88  (0.79-0.93) | 0.17  (0.12-0.24) | 0.36  (0.30-0.42) | 0.73  (0.58-0.85) | 41% | 0.05 |
| Self-Report  AQ50 | >=32 | ADOS-G | 204 | 0.74  (0.65-0.81) | 0.26  (0.17-0.39) | 0.65  (0.57-0.73) | 0.35  (0.23-0.49) | 57% | 0.00 |
|  |  | ADI-R | 290 | 0.75  (0.65-0.83) | 0.40  (0.33-0.47) | 0.39  (0.32-0.47) | 0.75  (0.65-0.83) | 52% | 0.15 |
| Informant-Report  AQ10 | >=6 | ADOS-G | 141 | 0.74  (0.63-0.82) | 0.25  (0.14-0.40) | 0.66  (0.56-0.75) | 0.33  (0.19-0.51) | 57% | -0.01 |
|  |  | ADI-R | 245 | 0.83  (0.73-0.90) | 0.26  (0.19-0.33) | 0.36  (0.29-0.43) | 0.75  (0.61-0.85) | 45% | 0.09 |
| Informant-Report  AQ50 | >=26 | ADOS-G | 153 | 0.94  (0.87-0.98) | 0.06  (0.01-0.17) | 0.65  (0.57-0.73) | 0.33  (0.09-0.69) | 63% | 0.00 |
|  |  | ADI-R | 260 | 0.97  (0.90-0.99) | 0.15  (0.10-0.21) | 0.37  (0.31-0.44) | 0.89  (0.70-0.97) | 43% | 0.12 |
| Informant-Report  AQ50 | >=32 | ADOS-G | 153 | 0.77  (0.67-0.85) | 0.17  (0.09-0.30) | 0.64  (0.54-0.72) | 0.28  (0.14-0.47) | 56% | 0.04 |
|  |  | ADI-R | 260 | 0.84  (0.75-0.91) | 0.40  (0.32-0.48) | 0.42  (0.35-0.50) | 0.83  (0.73-0.90) | 55% | 0.26 |

**Supplementary Table S2.** Diagnostic accuracy of the self-report and the informant report AQ10 and AQ50 as predictors of ASD caseness defined using the ADOS-G and ADI-R criteria. Standard ADOS-G criteria were used to identify ASD cases: patients scoring at least 2 on Communication , and at least 4 on Social Interaction, and with total scores across Communication and Social Interaction of at least 7, were classed as meeting criteria for ASD. The ADI-R includes a validated algorithm to identify individuals with narrowly defined autism: namely, at least 10 on social interaction, at least 8 on social communication, and at least 3 on repetitive behaviours and stereotyped patterns, with an age of onset of symptoms before 36 months. Individuals who meet criteria on all three domains were included in the ADI-R ASD group. For performance against clinical consensus diagnostic criteria, please see Table 2 in the main manuscript. Abbreviations: AQ=Autism-Spectrum Quotient, PPV=Positive Predictive Value, NPV=Negative Predictive Value. Youden J is defined as (Sensitivity + Specificity – 1). 95% confidence intervals are shown in parentheses for sensitivity, specificity, PPV and NPV.
